# COVID-19 mRNA Vaccine Induced Pericarditis With Large Pericardial Effusion Followed by New-Onset Arrhythmia

**DOI:** 10.7759/cureus.24699

**Published:** 2022-05-03

**Authors:** Krunalkumar Patel, Shivani Dalal, Daniel Tran, Akash Patel, Craig McMackin

**Affiliations:** 1 Internal Medicine, St. Mary Medical Center, Langhorne, USA; 2 Cardiology, St. Mary Medical Center, Langhorne, USA

**Keywords:** covid pericarditis, massive pericardial effusion, covid-19 mrna vaccine, covid-19, life threatening arrhythmia

## Abstract

Several cases of myocarditis and pericarditis have been reported after mRNA COVID-19 vaccination. Interestingly, most cases were seen in male adolescents and young adults, commonly after 3-4 days of the second dose of mRNA vaccine. The vaccine contains the viral spike glycoprotein in the nucleoside-modified mRNA of the coronavirus that activates the proinflammatory cascades and immunological pathways, which can cause myocarditis and pericarditis. Here we report a case of a middle-aged Caucasian male who presented with progressive dyspnea, fever, and chills seven days after the second dose of the COVID-19 vaccine. He was found to have pericarditis with massive hemorrhagic pericardial effusion, large bilateral pleural effusions, circulatory shock, and cardiac arrhythmia. The patient required pericardiocentesis and thoracentesis and was discharged home on antiarrhythmic and anti-inflammatory therapy. Our case report describes a correlation between mRNA COVID-19 vaccine and pericarditis, suggesting the importance of pharmacovigilance and reporting of adverse outcomes and encourages a high index of clinical suspicion in physicians to facilitate early diagnosis and interventions.

## Introduction

Vaccinations have been recognized as one of the most successful health measures to prevent widespread outbreaks of deadly diseases. More importantly, complications and side effects of vaccines are at a minimum, while benefits overwhelmingly outweigh any risks involved. However, it is vital to recognize any complications, especially given the growing number of individuals with vaccine hesitancy. One such complication involves myopericarditis. Different strains of vaccines historically have had different incidences of myopericarditis but are generally rare in patients of any age group [1]. A notable number of myopericarditis cases were described with the smallpox vaccine, and few were reported after influenza, yellow fever, and hepatitis B vaccine [1-4].

Since April 2021, cases of myocarditis and pericarditis have been reported to the Vaccine Adverse Event Reporting System (VAERS), occurring after mRNA COVID-19 vaccination. These cases were mostly seen in male adolescents and young adults aged 16 years or older and usually after receiving the second dose of the COVID-19 vaccine, typically appearing within four days of vaccination. Compared to the number of vaccine doses administered, these cases are rare. Most cases appear to be mild and often resolve without intervention [5]. Marshall et al. reported a case series of myopericarditis in adolescent males after the COVID-19 mRNA vaccine, and interestingly all these cases occurred after the second vaccine dose [6].

Here we describe a case of a 57-year-old male with pericarditis, large hemorrhagic pericardial effusion, and new-onset cardiac arrhythmia after the second dose of BNT162b2 (Pfizer-BioNTech) mRNA COVID-19 vaccination.

## Case presentation

A 57-year-old Caucasian male with a history of COVID-19 infection five months prior and chronic GERD, not requiring medications, presented to the emergency department to evaluate progressively worsening dyspnea with minimal exertion and intermittent fevers, chills, and fatigue. The patient reported persistent left-sided burning chest discomfort associated with dyspnea and night sweats for the past two weeks. His pain was also aggravated by coughing, leaning forward, and being relieved in the supine position. Interestingly, he received his second dose of BNT162b2 (Pfizer-BioNTech) mRNA COVID-19 vaccine almost a week before symptom onset. After the first dose of the COVID-19 vaccination, the patient did not experience any side effects such as fever, headaches, rash, or myalgias. On presentation, his blood pressure was 116/73 mmHg, heart rate 97 beats per minute, oxygen saturation 93 % while breathing ambient air, and body temperature 36.9°C.

Electrocardiogram demonstrated normal sinus rhythm and no evidence of acute ischemia. The chest x-ray showed enlargement of the cardiac silhouette and bilateral pleural effusions. Testing for common respiratory viruses by RT-PCR was negative. Labs revealed elevations in transaminases, alkaline phosphatase, D-dimer, ferritin, and C-reactive protein. Complete blood count with eosinophil differentials was normal, and interestingly, high-sensitivity troponin I was unremarkable (3 ng/L) on repeated measurements one hour apart.

**Figure 1:**
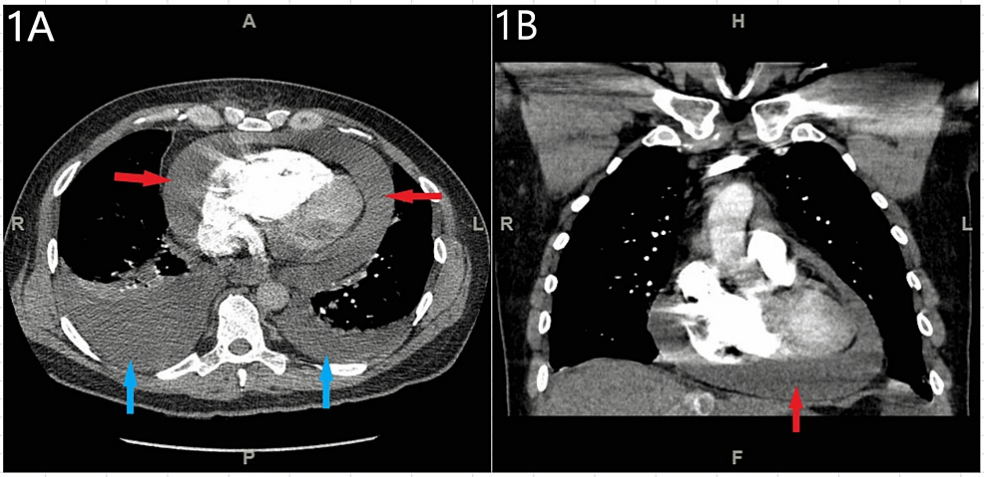
CT angiography of the chest showed large pericardial effusion (red arrows), large right and moderate left pleural effusions (blue arrows). Figure 1A - axial view, Figure 1B - coronal view

CT angiography of the chest/abdomen/pelvis was notable for a large pericardial effusion with reflux of contrast into the inferior vena cava and hepatic veins, large right and moderate left pleural effusions, moderate ascites throughout the abdomen and pelvis (Figure 1).

**Figure 2:**
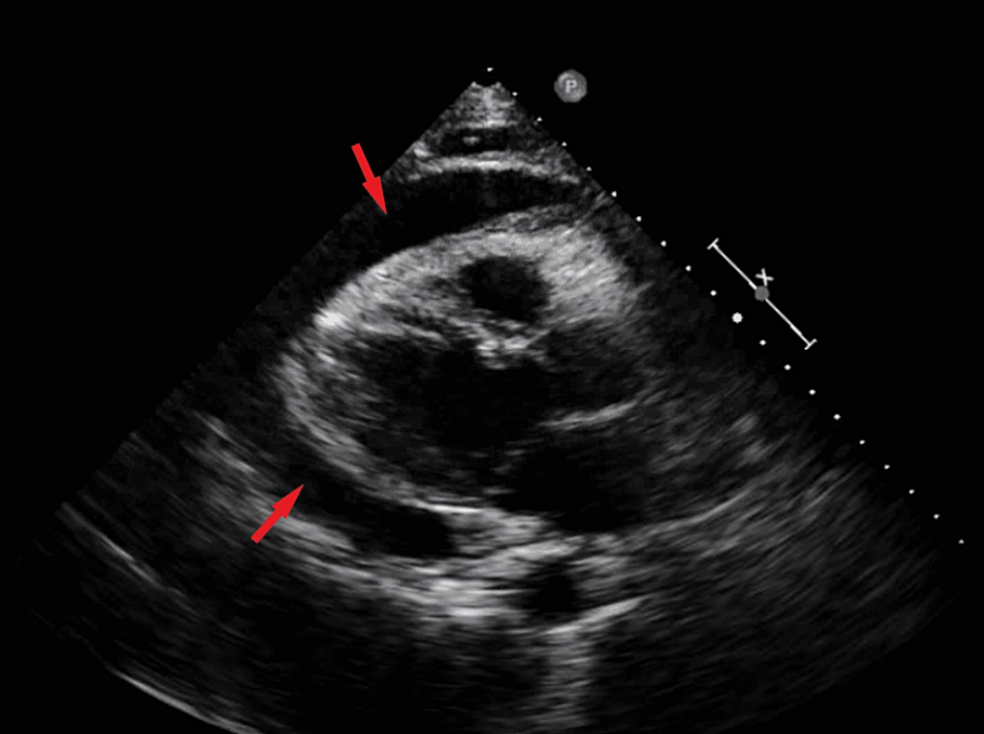
Transthoracic echocardiogram showed large pericardial effusion (red arrows).

Further, the patient developed rapid atrial flutter with associated hypotension requiring urgent echocardiography, which confirmed large pericardial effusion. The left ventricular function was preserved, no significant valvular defects were noted, and there was no evidence of tamponade physiology (Figure 2).

However, given his rapidly accumulating large pericardial effusion and persistent hypotension, 800cc of hemorrhagic fluid was drained by pericardiocentesis. Thoracentesis was also performed for his large pleural effusions, and 900cc and 700cc of fluid were removed from his left and right pleural spaces, respectively. Pleural and pericardial cytological analysis was positive for mesothelial and inflammatory cells and negative for malignancy. The patient was initiated on Colchicine and Ibuprofen for pericarditis. His hospital course was complicated by new-onset atrial fibrillation requiring the initiation of amiodarone. A repeat echocardiogram was performed, which demonstrated a trivial amount of pericardial fluid. The patient was discharged home after resolving his symptoms and optimizing his medical therapy. On follow-up with his cardiologist, no atrial arrhythmias were noted. A repeat chest CT was performed after three weeks of discharge, demonstrating no recurrence of pericardial effusion and complete resolution of bilateral pleural effusions.

## Discussion

This case report highlights a correlation between the second dose of BNT162b2 (Pfizer-BioNTech) mRNA COVID-19 vaccine and severe hemorrhagic pericardial effusion and cardiac arrhythmia in an otherwise healthy patient with a history of distant COVID-19 infection. It is biologically plausible that the vaccines could trigger massive immune activation, leading to severe inflammation of the heart and a multitude of organ system involvement [7,8]. SARS-CoV-2 mRNA vaccines do not contain live viruses or DNA but have nucleoside-modified mRNA, which encodes the viral spike glycoprotein of the coronavirus. In certain individuals, such mRNA vaccine might be detected as an antigen by the immune system, resulting in the activation of proinflammatory cascades and immunological pathways, which may cause myocarditis and pericarditis as part of a systemic reaction [7,8].

Heart inflammation can develop rapidly or progress slowly, with symptom severity ranging from none to severe symptoms. If inadequately managed, complications can progress to severe arrhythmias, blood clots, or heart failure. Most fatal complications can include cardiac tamponade, resulting from an excess fluid collection in the pericardial cavity-causing circulatory shock, which can be lethal enough to cause death [9]. Classic symptoms of pericarditis involve sharp, stabbing chest pain that comes on suddenly, along with occasional fevers, weakness, and shortness of breath. Following smallpox vaccination in the past, acute myocarditis was commonly associated with eosinophilic myocarditis [9]. Our case presentation did not observe peripheral eosinophilia or hypersensitivity reactions; however, our patient had elevated inflammatory markers such as ferritin and c-reactive protein.

Systemic reactogenicity occurred more commonly after the second dose of the COVID-19 vaccine and in younger patients [10,11]. Even without significant lung involvement, cases of acute myocarditis have been reported after COVID-19 vaccination, which suggests a viral triggered immune-mediated injury [10]. Marshall et al. reported a case series of patients with mild myocarditis after the second dose of the COVID-19 vaccine [6]. Similar cases have been reported in Israel, where myocarditis occurred predominantly in a population of young males up to age 30 who received the Pfizer-BioNTech COVID-19 vaccine [12]. A case report from Italy described a 56-year-old male patient with a prior COVID-19 infection who developed myocarditis after mRNA COVID-19 vaccination [13]. Kaul et al. reported two cases of acute myocarditis within three days of mRNA COVID-19 vaccination, and both recovered after conventional therapy for myocarditis [14]. The majority of data on COVID 19 vaccine-associated cardiac involvement have been mild in severity and usually seen among young males [11,14]. In contrast, our patient was a middle-aged gentleman with severe pericarditis, which progressed to hemorrhagic pericardial effusion and cardiac arrhythmias with associated multi-organ system involvement.

Notably, it can be hypothesized that in some cases, acute myocarditis and pericarditis can present only mild symptoms, which patients can manage with over-the-counter medications without seeking any medical advice. The existing database reports fewer cases after COVID-19 vaccination compared to the massive number of doses already administered, strengthening the importance of continuous post-marketing surveillance.

## Conclusions

In conclusion, given the paucity of cases, the risk of pericarditis from COVID-19 vaccines is negligible. Identification of individuals at an increased risk of autoimmune reactions before mRNA vaccination may allow reasonable precautions to be taken. Additionally, a recent vaccination history can help in the differential diagnosis in cases involving specific symptoms. Continued monitoring and reporting to the VAERS are strongly recommended, as such reports would raise clinicians’ awareness to suspect and treat these cases at early onset.
